# A novel way to establish fertilization recommendations based on agronomic efficiency and a sustainable yield index for rice crops

**DOI:** 10.1038/s41598-017-01143-2

**Published:** 2017-04-24

**Authors:** Chuang Liu, Yi Liu, Zhiguo Li, Guoshi Zhang, Fang Chen

**Affiliations:** 1 Key Laboratory of Aquatic Botany and Watershed Ecology, Wuhan Botanical Garden Chinese Academy of Sciences, Wuhan, 430074 China; 2 University of Chinese Academy of Sciences, Beijing, 100049 China; 3 China Program of International Plant Nutrition Institute, Wuhan, 430074 China

## Abstract

A simpler approach for establishing fertilizer recommendations for major crops is urgently required to improve the application efficiency of commercial fertilizers in China. To address this need, we developed a method based on field data drawn from the China Program of the International Plant Nutrition Institute (IPNI) rice experiments and investigations carried out in southeastern China during 2001 to 2012. Our results show that, using agronomic efficiencies and a sustainable yield index (SYI), this new method for establishing fertilizer recommendations robustly estimated the mean rice yield (7.6 t/ha) and mean nutrient supply capacities (186, 60, and 96 kg/ha of N, P_2_O_5_, and K_2_O, respectively) of fertilizers in the study region. In addition, there were significant differences in rice yield response, economic cost/benefit ratio, and nutrient-use efficiencies associated with agronomic efficiencies ranked as high, medium and low. Thus, ranking agronomic efficiency could strengthen linear models relating rice yields and SYI. Our results also indicate that the new method provides better recommendations in terms of rice yield, SYI, and profitability than previous methods. Hence, we believe it is an effective approach for improving recommended applications of commercial fertilizers to rice (and potentially other crops).

## Introduction

Rice (*Oryza sativa* L.) is one of the most important cereal crops in the world, and the one most widely planted in China. The rice crop yield amounted to 20.8 million tons, accounting for 33.5% of total food crop production in China in 2015^1^. However, owing to continuous high production and fertilizer inputs, the fertilization efficiency and farmland environmental quality in the country’s rice-growing regions have declined in recent decades^2, 3^. Thus, increases in grain production will incur more resource and environmental costs unless the sustainability of nutrient use is increased in China^4^. Much research has shown that the farmland environmental quality is affected by unbalanced or excessive fertilization^5^, which can cause imbalances in soil nutrients and exacerbate their losses while increasing the agricultural non-point pollution^6, 7^. Thus, scientifically robust and convenient methods for establishing fertilizer recommendations are required that can both improve farmland nutrient recycling and reduce risks of environmental pollution.

Recommended fertilization and farmland nutrient management methods are mainly based on soil testing and crop yield responses^8, 9^. Soil testing methods, as exemplified by the soil fertility index method to determine optimum fertilizer applications and achieve target yields, have been widely used and promoted^10, 11^. However, methods based on crop yield responses, notably the Nutrient Expert system for hybrid maize, are gaining increasing popularity^12, 13^. In China, formula fertilization via soil testing has predominated since the 1980s, but this method is economically incompatible with a smallholder farming system, because testing is prohibitively expensive for farmers with limited budgets^14, 15^. Moreover, variations in climates and soil types lead to variations in indigenous nutrient-supplying capacities and nutrient availabilities obtained from soil tests. Hence, a new fertilizer recommendation approach is needed to address the growing imbalances between nutrient supplies and demands in Chinese rice cropping systems.

An important variable used to formulate fertilizer recommendations in the Nutrient Expert system mentioned above is the agronomic efficiency (the increase in yield of a crop per unit of a given nutrient supplied). We hypothesized that correlations between agronomic efficiency with yield responses (the differences in yield between controls plots that receive ample nutrients and corresponding plots that receive all but one of the added nutrients), economic cost/benefit ratios, and nutrient-use efficiencies could be used to develop a more convenient approach.

The first objective of the study presented here was to test the robustness of these correlations, using ranked levels of agronomic efficiency of nitrogen (N), phosphorus pentoxide (P_2_O_5_) and potassium oxide (K_2_O) (AEN, AEP and AEK, respectively). The correlations were then used to: improve recommended application rates of N, P, and K fertilizers for rice cultivation (which may be applicable to other grain crops) and analyze relationships between rice yield responses and manurial value. Finally, an innovative method for formulating fertilizer recommendations was developed, based on agronomic efficiencies and a previously published sustainable yield index (SYI; derived by subtracting the estimated standard deviation of yield associated with a practice from the estimated mean over a number of years then dividing by the observed maximum yield)^16^.

## Results and Discussion

### Yield response under the AE gradation index

**Figure 1:**
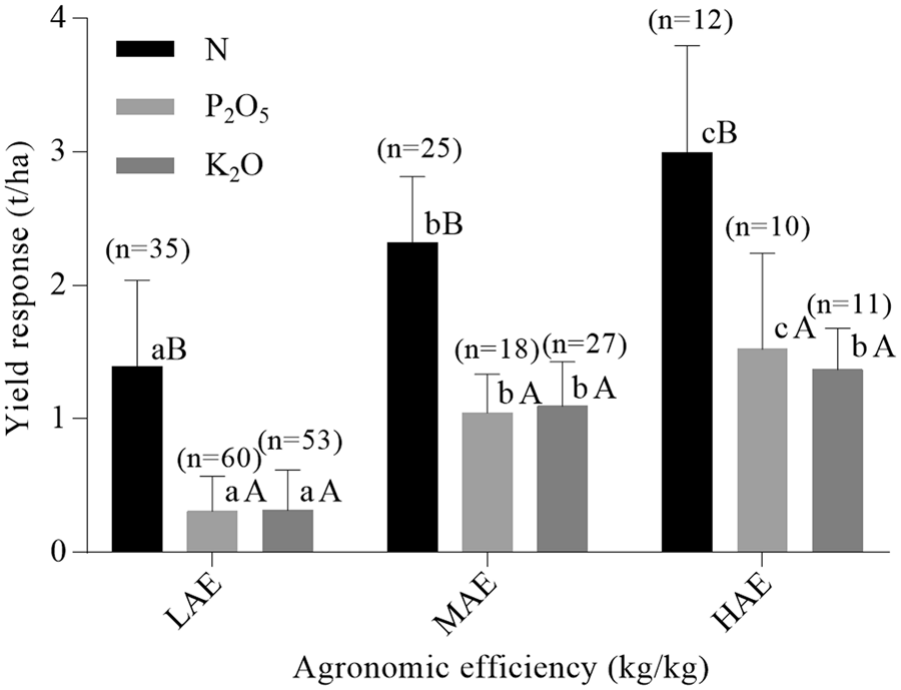
Yield responses (mean ± SD) to N, P_2_O_5_ and K_2_O fertilizer applications at low (LAE), medium (MAE), and high agronomic efficiency (HAE). Within each level, different capital letters indicate a significant difference at *P* < 0.01, whereas among the agronomic efficiency (AE) levels differing lower-case letters indicate a significant difference at *P* < 0.05.

Yield responses are among the most important variables for evaluating fertilizer efficiency in farmland systems, because they reflect the cycling of soil nutrients in agroecosystems and gaps between attainable and nutrient-limited yields. Significant negative correlations have been found between yield responses of both rice and maize crops, and indigenous soil nutrient supply capacities in China^17, 18^. The present study revealed significant differences in yield responses to N, P, and K fertilizers both among and within the three AE levels (Fig. 1). As expected, high agronomic efficiency (HAE) was associated with high N, P, and K yield responses (and hence high rice yields)^19^. For example, the yield response to N at HAE was *c*. 3.0 t/ha, which exceeded the corresponding values at medium agronomic efficiency (MAE) and low agronomic efficiency (LAE).

### Variations in relative yield

**Figure 2:**
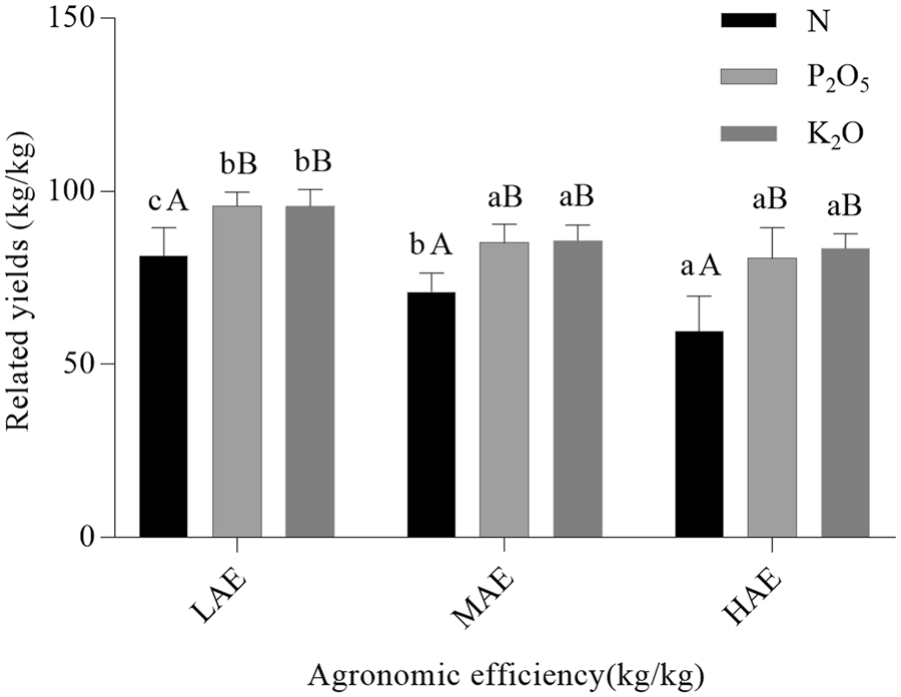
The relative yield (mean ± SD) of N, P_2_O_5_ and K_2_O fertilizer applications at low (LAE), medium (MAE), and high agronomic efficiency (HAE). Within each level, different capital letters indicate a significant difference at *P* < 0.01, whereas among the agronomic efficiency (AE) levels different lower-case letters indicate a significant difference at *P* < 0.05.

As shown in Fig. 2, there was a significant negative correlation between the relative yield (RY; the proportion between nutrient-limited yield and attainable yield with optimal fertilization) of rice and AEN levels (RY was 81.4, 71.0 and 59.5% at high, medium and low levels, respectively), and slight (non-significant) correlations between RY and both AEP and AEK levels. Similarly, a negative correlation between RY of early rice and soil alkali-N content has been recorded at a field site in China^20^, and an inverse relationship between soil-available P and K. Hence, it is plausible that soil alkali-N content and soil available P and K contents were higher at the HAE level than at either the MAE or LAE levels. The results also show that relative yields obtained from P_2_O_5_ and K_2_O applications were higher than those obtained from N applications, suggesting that rice is more sensitive to N deficiency than either P or K deficiency. Taken together, the results presented here justify the greater attention paid to the distribution and supply of N in rice cultivation.

### Variations in fertilizer contribution rates

**Figure 3:**
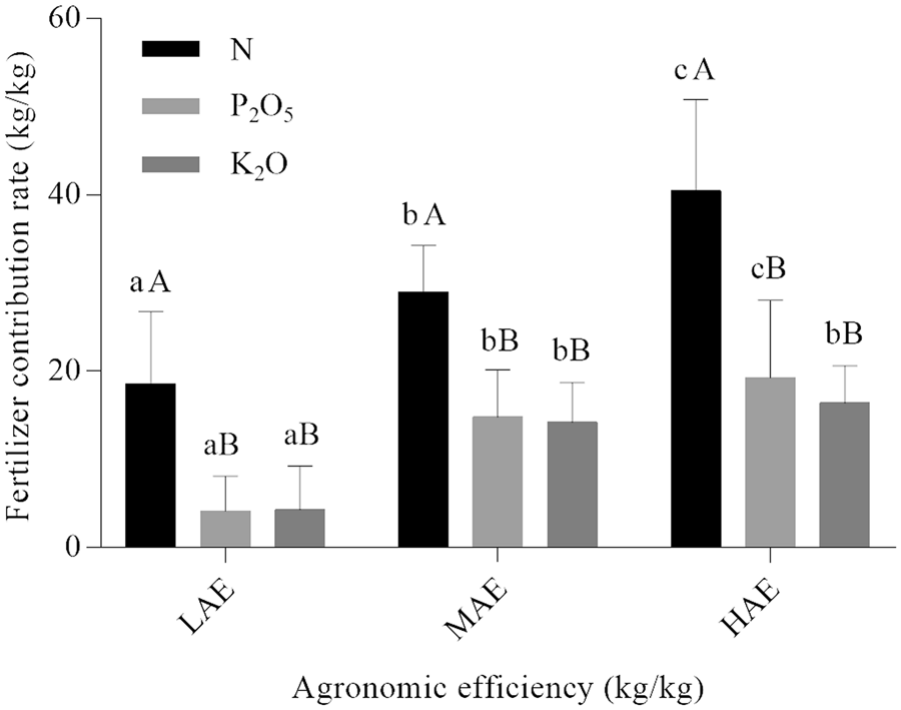
The Fertilizer contribution rate (mean ± SD) of N, P_2_O_5_ and K_2_O at low (LAE), medium (MAE), and high agronomic efficiency (HAE). Within each level, different capital letters indicate a significant difference at *P* < 0.01, whereas among the agronomic efficiency (AE) levels differing lower-case letters indicate a significant difference at *P* < 0.05.

The fertilizer contribution rate is defined here as the increase in yield per unit of a given nutrient in a fertilization treatment. The highest FCRs of N, P_2_O_5_, and K_2_O derived from our dataset were 40.5, 19.3, and 16.4 kg/kg, respectively. The results shown in Fig. 3 also indicate that FCRs of N were higher than those of P_2_O_5_ and K_2_O. Similar rankings (but lower absolute values) have been previously reported in a study indicating that the optimum FCRs of N, P_2_O_5_, and K_2_O were respectively 13.9, 5.9, and 9.9 kg/kg for rice cultivation in China^21^. An FCR of N of 29.6 kg/kg in a well-managed Chinese rice cultivation system with a low-N use or low-N soil supply has also been reported^22^. The FCR of N significantly differed between the LAE (18.6%), MAE (30.0%), and HAE (40.5%) levels (P < 0.01). In addition, there was a significant positive correlation between FCR and AEP levels, and FCR increased with increases in AEK (Fig. 3).

### Evaluation of economic benefits

To assess the economic benefits of fertilization at the three AE levels (with applications of urea, potassium chloride and calcium superphosphate), costs, benefits and net benefits (across the trials used in the analysis) were calculated using the following equations^23^:

$${\rm{Total}}\,{\rm{fertilizer}}\,{\rm{cost}}={\rm{N}} \mbox{-} \mathrm{fertilizer}\,{\rm{cost}}+{\rm{P}} \mbox{-} \mathrm{fertilizer}\,\mathrm{cost}+{\rm{K}} \mbox{-} \mathrm{fertilizer}\,\mathrm{cost};$$

$${\rm{Gross}}\,{\rm{revenue}}={\rm{Grain}}\,{\rm{yield}}(\mathrm{kg}/\mathrm{ha})\times {\rm{Rice}}\,{\rm{market}}\,{\rm{price}}(\mathrm{yuan}/\mathrm{kg});$$

$${\rm{Net}}\,{\rm{benefit}}={\rm{Gross}}\,{\rm{revenue}}-{\rm{Total}}\,{\rm{fertilizer}}\,{\rm{cost}}$$

**Figure 4:**
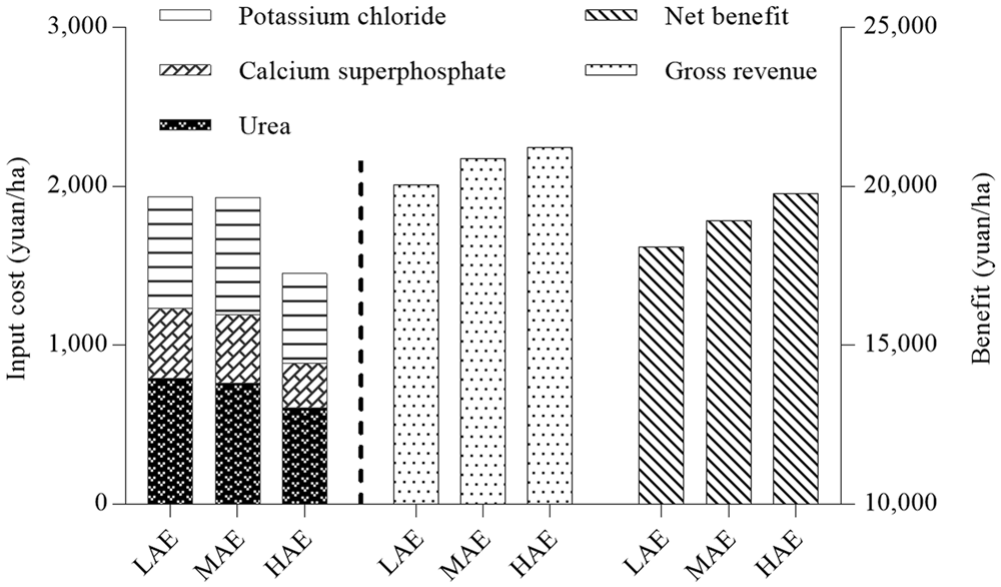
Estimated input costs and benefits of fertilization in rice cultivation at low (LAE), medium (MAE), and high agronomic efficiency (HAE). The price of rice was fixed at 2.7 RMB yuan/kg; prices for N, P_2_O_5_, and K_2_O were fixed at 1.8, 3.0, and 0.7 RMB yuan/kg, respectively.

The data suggest that increasing AE reduces costs and increases benefits (Fig. 4). Urea and potassium chloride accounted for 77.2–80.5% of the total fertilizer costs at each AE level, and calcium superphosphate for the rest (19.5–22.8%). Both the gross revenue and net benefit increased as AE increased. In addition, the total fertilizer cost was lower (and the proportional contributions of urea and potassium chloride to total costs were higher) for HAE than for either MAE or LAE. However, we would also provide indications of the statistical variation in either the figure or figure and text.

### Construction and framework of the fertilizer recommendation method

**Figure 5:**
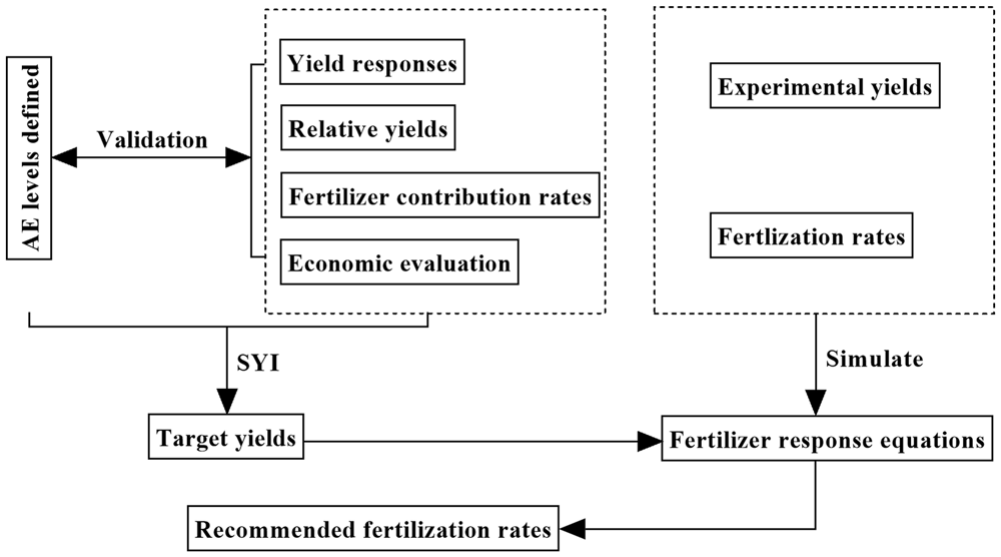
Framework of the new fertilizer recommendation method based on agronomic efficiency (AE) and sustainable yield index (SYI).

Various approaches for formulating fertilization recommendations based on soil tests and crop yield responses have been developed^24–27^. Here, we present another approach (for rice crops) based on two important indices, SYI and AE, validated using yield responses, relative yields, fertilizer contribution rates, target yields and economic benefits calculated from the extensive dataset described in the *Materials* and *Methods* section. Fertilizer response equations were developed to model the relations between the experimental yields and fertilization rates, then recommended fertilization rates were derived from the target yields and fertilizer response equations, as illustrated in Fig. 5.

The novel method based on AE and SYI involves the following four steps: (1) calculation of average yields under the optimal (OPT) treatment (defined as described in the *Materials* and *Methods* section, or some other appropriate manner) and SYI at each of the AE levels; (2) linear regression analysis of the derived OPT yield and SYI values; (3) determination of target yield ranges from the results of Step 2; and (4) calculation of recommended N-, P-, and K-fertilization rates using the mean target yields derived in Step 3.

Those steps are described in detail in the following sections.

#### Step 1: Calculation of Average OPT Yield and SYI

Mean OPT yield and SYI values for at each specified AE level (here, LAE, MAE, and HAE). However, for use in the linear regression modeling, the entire datasets require processing. Therefore, a crucial element of Step 1 is ranking the data for calculation according to the AE values.

#### Step 2: Regression Analysis

**Figure 6:**
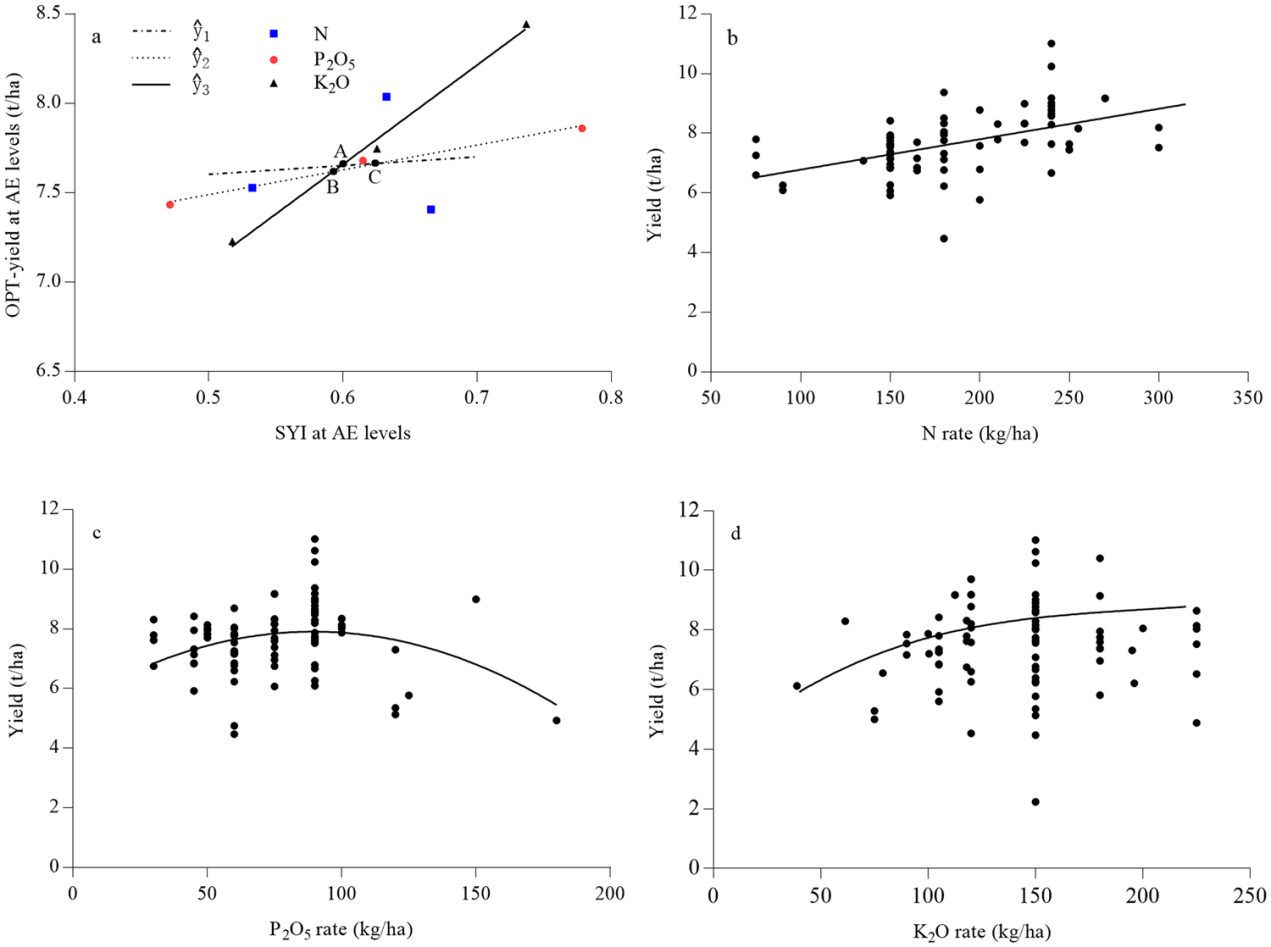
Optimum yield (*ŷ*) at agronomic efficiency (AE) and experimental yield (*y*_*1*_ *, y*_*2*_ *, y*_*3*_) modelled as functions of the sustainable yield indices (SYI) (*Ŝ*), N (*x*_*1*_), P_2_O_5_ (*x*_*2*_) and K_2_O (*x*_*3*_).

**(a)** Equations linking average OPT yield (*ŷ*) and SYI (*Ŝ*_*1*_) at each AE level are generated by linear regression. Here, as illustrated in Fig. 6a, the following three equations (where the subscripts 1, 2 and 3 refer to AE levels 1, 2 and 3, respectively) were generated, using Microsoft Excel 2016:

1 $${\hat{y}}_{{\rm{1}}}={\rm{0.4869}}\times {\hat{S}}_{{\rm{1}}}+{\rm{7.3608}}$$

2 $${\hat{y}}_{{\rm{2}}}={\rm{1.3848}}\times {\hat{S}}_{{\rm{2}}}+{\rm{6.7985}}$$

3 $${\hat{y}}_{{\rm{3}}}={\rm{5.5519}}\times {\hat{S}}_{{\rm{3}}}+{\rm{4.3295}}$$

**(b)** Curvilinear equations are then generated to model the relationship between fertilizer doses and experimentally recorded yields of rice crops. Here, as illustrated in Fig. 6b–d, the derived equations (where *x*_*1*_, *x*_*2*_, and *x*_*3*_ refer to N, P_2_O_5_ and K_2_O, respectively), are:

4 $${y}_{{\rm{1}}}={\rm{0.102}}{x}_{{\rm{1}}}+{\rm{5.7656}}({{\rm{R}}}^{2}=0.2492,{\rm{P}} < 0.01,{\rm{n}}=72)$$

5 $${y}_{{\rm{2}}}={\rm{0.0003}}{x}_{{\rm{2}}}^{{\rm{2}}}+{\rm{0.0537}}{x}_{{\rm{2}}}+{\rm{5.5086}}({{\rm{R}}}^{2}=0.0977,{\rm{P}} < 0.01,{\rm{n}}=89)$$

6 $${y}_{{\rm{3}}}=5E\,-\,07{x}_{{\rm{3}}}^{{\rm{3}}}\,-\,{\rm{0.0003}}{x}_{{\rm{3}}}^{{\rm{2}}}+{\rm{0.0645}}{x}_{{\rm{3}}}+{\rm{3.7877}}({{\rm{R}}}^{2}=0.035,{\rm{P}} < 0.01,{\rm{n}}=91)$$

The equations shown in Fig. 6 proved satisfactory for modeling the data from the IPNI experiments (described in the *Materials* and *Methods* section).

#### Step 3: Target Yields

In this new fertilizer recommendation approach, the range of target yields is based on the intersection of the linear equations 1, 2 and 3 (plotted as A, B and C in Fig. 6a), and estimated (using the IPNI dataset) at 7.62~7.67 t/ha.

#### Step 4: Fertilization Rates

Finally, N, P and K fertilization rates required to meet target yields determined in Step 3 are obtained using eqs 4, 5, and 6, respectively. Here, the average N, P_2_O_5_, and K_2_O fertilization rates obtained were 186, 60, and 96 kg/ha, respectively (Fig. 6a–c).

The key element of this fertilizer recommendation method is ranking agronomic efficiency to calculate sustainable yield indices in order to determine target yield ranges. As it relies on data concerning aboveground plant parts it should be more convenient than methods based on relationships between indices derived from soil tests and crop yield responses^28, 29^. N, P_2_O_5_, and K_2_O fertilization rates for rice reportedly ranged from 82.5 to ~247.5, 30.0 to ~90.0, and 45.0 to ~150.0 kg/ha, respectively, in Hubei province in 2007–2010^30^. The recommended rates generated by our method are within these ranges. The previously mentioned Nutrient Expert System for Hybrid Maize^12^ is based on a similar approach to our method (involving use of yield responses, AE, relative yields, and indigenous soil nutrient supply capacities to obtain robust estimates of yields and suitable fertilizer rates for maize). However, an inherent drawback is its inability to evaluate sustainable yields of a crop, and it could be improved by integrating SYI in its validation protocols. We believe our new method is simpler, more convenient, and may be advantageous for maintaining rice yields, sustainability, and profitability. Thus, it may offer an effective approach for improving recommendations for commercial fertilizer applications in China (which have often been previously generated using model equations calculated by agricultural extension workers based on various farmers’ rice yields or an arbitrary 10% increase in yields).

## Conclusion

A new method for formulating recommended fertilizer rates for rice crops is proposed. It is derived from comprehensive analysis of the relationships among agronomic efficiency, sustainable yield index, yield response, relative yield, fertilizer contribution rate, and economic benefits using datasets collected from 251 farmer’s fields in southeast China during 2001 to 2012. The method generated recommended mean N, P_2_O_5_ and K_2_O fertilization rates for rice crops in the region of 186, 60 and 96 kg/ha, respectively. We found a significant positive linear relationship between yields under optimal fertilization and a sustainable yield index at identified agronomic efficiency levels, as well as significant linear or nonlinear relationships between experimental yields and N, P_2_O_5_, and K_2_O fertilizer rates. A new agronomic efficiency gradation index is proposed based on the 251 farm field experiments in southeast China, and fertilizer recommendation equations for rice are recommended.

## Materials and Methods

### Data and experimental sites

**Figure 7:**
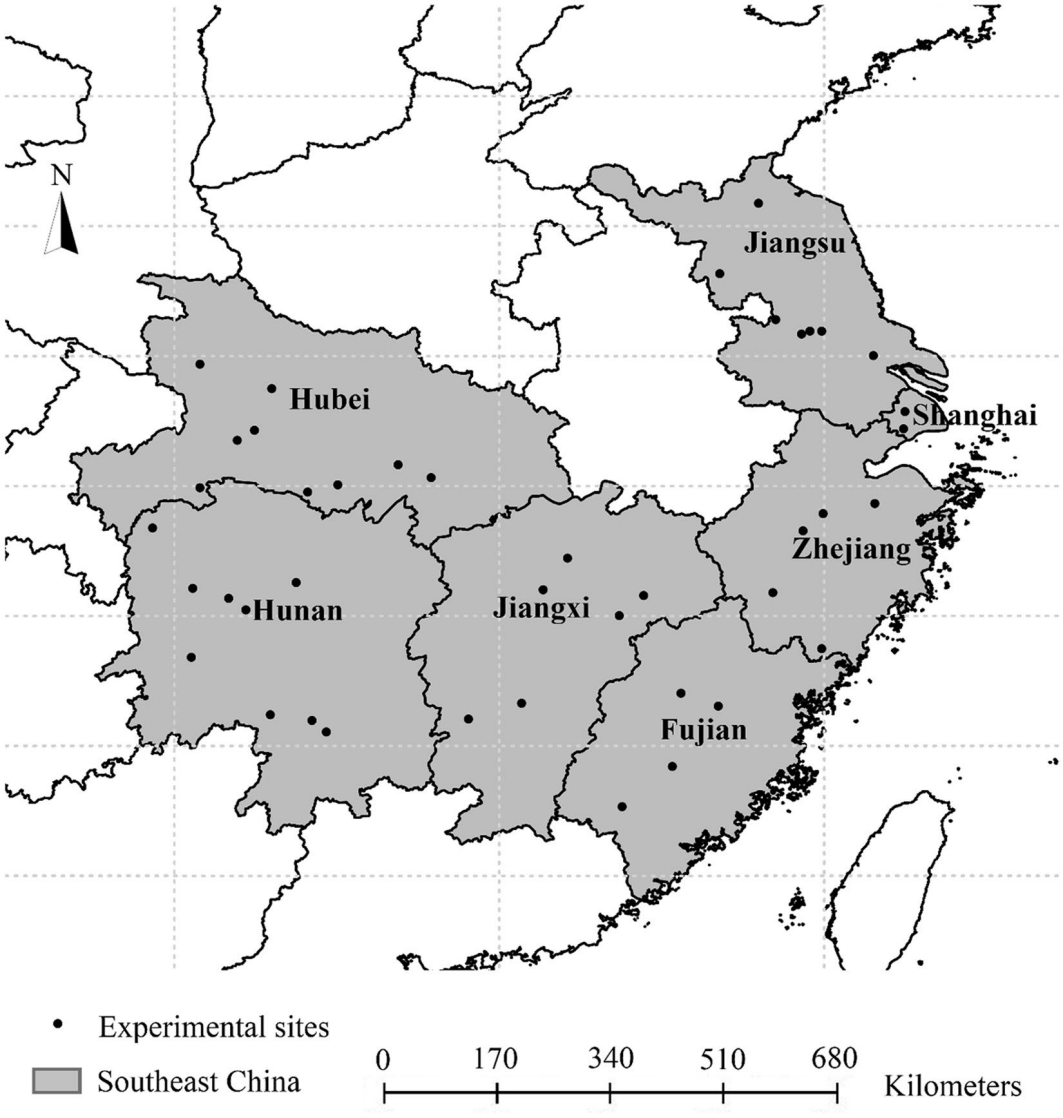
Locations of the experimental sites of rice projects in China that provided the datasets used in this study (plotted using ArcGIS v.10.0 software, URL: http://www.esri.com).

The datasets used in this study were obtained from the field experiments that conducted from 2001 to 2012 in the cooperative projects of the International Plant Nutrition Institute (IPNI) China Program. The experimental sites were located in China’s main rice-production regions in seven south-eastern provinces - Jiangsu, Zhejiang, Shanghai, Jiangxi, Fujian, Hunan, and Hubei provinces, which collectively include *ca*. 50% of the national rice planting area (Fig. 7). At each site the field experiments usually included at least four major treatments: NPK (OPT; close to optimal applications of N, P and K), OPT-N, OPT-P, and OPT-K. The N, P and K application rates in the OPT treatments (providing indications of the maximum grain yield at each site per year) were based on the researchers’ former trial results and numerous years of experimental experience. Each field experiment was arranged in a randomized complete block design, with three or four replications, using 30–50 m^2^ plots. Rice varieties, fertilizer sources, and planting management practices were identical to those used by local farmers.

### Determination of agronomic efficiency gradation indices

**Figure 8:**
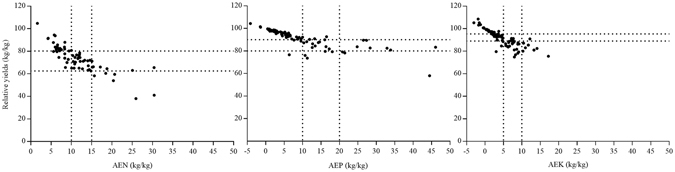
Relationships between relative yield (the proportion between nutrient-limited yield and attainable yield with optimal fertilization) and agronomic efficiencies of N, P and K (AEN, AEP and AEK, respectively) in the rice cultivation field experiments in southeast China that provided the data used in this study.

Agronomic efficiency (AE) is the increase in yield of economically valuable parts of a crop (here grain) from per unit of a given nutrient applied. It is an important parameter for evaluating the proportions of added nutrients that are absorbed and used by crops. AEN, AEP, and AEK were calculated using data obtained from 251 field experiments over an 11-yr period (2001 to 2012) in the program mentioned above. Using an improved Cate–Nelson method^31, 32^, each AE was divided into three levels: low, medium and high. These three levels were, respectively: <10, 10–15 and >15 kg/kg for AEN (mean, 11.06 kg/kg); <10, 10–20 and >20 kg/kg for AEP (mean, 8.93 kg/kg) and 5, 5–10 and >10 kg/kg for AEK (mean, 5.03 kg/kg) (Fig. 8).

### Data calculations and analysis

Standard procedures were used to analyze and calculate the yield response, fertilizer contribution rate, relative yield, gross income, and cost and net profits^8–12, 20^. The sustainable yield index (SYI) used was previously reported^16^. Plants subjected to each treatment in the field sites in China were randomly selected for sampling (in a standardized way) to determine grain yield at harvest time. The data were subjected to one-way ANOVA (analysis of variance) and the differences between groups were compared with the LSD test. These analyses were performed using SPSS v.18.0 software. These figures (Figs 1–6, 8) were plotted using GraphPad Prism v. 7.0 software, URL: http://www.graphpad.com.
